# Morphological and Molecular Identification of *Fusarium ipomoeae* as the Causative Agent of Leaf Spot Disease in Tobacco from China

**DOI:** 10.3390/microorganisms10101890

**Published:** 2022-09-22

**Authors:** Hancheng Wang, Yiting Li, Wenhong Li, Liuti Cai, Jianyu Meng, Gen Xia, Junliang Yin, Xi Liu

**Affiliations:** 1 Guizhou Provincial Academician Workstation of Microbiology and Health, Guizhou Academy of Tobacco Science, Guiyang 550081, China; 2 College of Agriculture, Yangtze University, Jingzhou 434025, China; 3 Guizhou Institute of Plant Protection, Guizhou Academy of Agricultural Sciences, Guiyang 550006, China

**Keywords:** *Fusarium*, tobacco, morphology, pathogenicity, phylogenetic analysis

## Abstract

Tobacco (*Nicotiana tabacum* L.), which creates jobs for 33 million people and contributes two trillion dollars’ tax annually, is one of the most important economic plants globally. However, tobacco is seriously threatened by numerous diseases during production. Previously, the field survey of tobacco diseases was conducted in the Guizhou and Guangxi provinces, the two main tobacco-producing areas in China. A serious leaf spot disease, with a 22% to 35% incidence, was observed in farming plants. In order to determine the causal agents, we collected the disease samples and isolated the pathogenic fungi. The pathogen was identified as *Fusarium* *ipomoeae*, based on the morphological characteristics and phylogenetic analysis. Pathogenicity tests showed that *F. ipomoeae* could induce tobacco leaf spot and blight. To our knowledge, this is the first report worldwide of *F. ipomoeae* causing leaf spots and stems on tobacco. Our study reveals the serious consequences of *F. ipomoeae* on tobacco filed production and provides information for future diagnosis and management of the *Fusarium* disease.

## 1. Introduction

Tobacco (*Nicotiana tabacum* L.) is one of the most crucial economic crops all over the world [1]. Globally, according to the estimates of tobacco industry, about 33 million people participate in tobacco planting, product manufacturing, distribution, and retailing [2]. Meanwhile, global revenues from the tobacco industry are estimated at 2 trillion dollars a year [3]. In China, tobacco was planted in more than 14,000 km^2^, and its yield of leafage reached 31.32 million tons in 2011 [4]. Its accounts for more than 39.6% of total global tobacco production [5], in which, farming is especially prevalent in Southwest China [6]. Among the main supplier provinces, Guizhou province produces nearly 30% of the total Chinese tobacco crop and ranks as the second-most tobacco-producing areas [7].

It is well-known that tobacco suffers from various fungal and oomycete pathogens during its whole growing season, such as *Fusarium* spp., *Collectotrichum gloeosporioides*, *Alternaria alternata*, *Botrytis cinraea*, etc. [6,7]. Among them, *Fusarium* spp., which can cause tobacco leaf, root, and stem diseases, have been a serious problem threatening to tobacco production in many countries [8]. The incidence of *Fusarium* spp. has increased considerably in recent years [6,7,8]. Furthermore, the mycotoxins and secondary metabolites produced by the *Fusarium* species in diseased leaves may be harmful to the health of humans [9]. Therefore, *Fusarium* identification was of particular importance for the effective management of tobacco diseases.

In August 2021, the field survey of tobacco disease was conducted in Zheng’an and Fenggang in Guizhou, Shanglin. The three main tobacco-producing areas in China are located in Guangxi. Tobacco plants with leaf spots and blights were frequently observed in the farming fields, with a 22 to 35% disease incidence. In order to determine the causal agents, provide information for future diagnosis, and help the management of this disease, the disease samples were collected, and the pathogenic fungi were isolated. Morphology characterization and multi-gene locus phylogenetic analysis were performed. Furthermore, the pathogenicity was also tested following the Koch postulates.

## 2. Materials and Methods

### 2.1. Isolation

In August 2021, diseased plants of tobacco were collected from three main producing counties (Zheng’an and Fenggang in Guizhou and Shanglin in Guangxi). For fungal isolation, the diseased tissues were cut into several small segments and placed on potato dextrose agar (PDA, Difcohai) at 25 °C in darkness for 3 to 5 days. To obtain pure cultures, hyphal tips from developed colonies were transferred to fresh PDA plates three times [10]. The isolates were inoculated to PDA test tube slants and stored at 4 °C [11]. The strain was deposited at Yangtze University, Jingzhou, Hubei, China.

### 2.2. Morphology

The edge of the colony was cut into 6 mm diameter plates with a sterile punch, and the mycelia plug was transferred to a 90 mm PDA plate and grown for 7 days at 25 °C in darkness. Then, the colony morphology was evaluated and visualized. For observing the morphological features of conidia, the marginal hyphae were transferred to carnation leaf-piece agar (CLA) medium (the sterilized carnation leaves were placed in water agar medium) and cultured at 22 °C under a light/dark period of 12/12 h [12]. After 7–10 days, conidia and chlamydospores were mounted in sterile water for microscopic observation using a Nikon ECLIPSE Ni–U microscope equipped with a Nikon DS–Ri2 digital camera (Tokyo, Japan) [12].

### 2.3. DNA Extraction and PCR Amplification

Genomic DNA was extracted from fresh mycelium grown on PDA using a method modified from Cenis [13]. Five loci, including the 5.8S nuclear ribosomal RNA gene with the two flanking internal transcribed spacers (ITS), translation elongation factor 1 alpha (*EF–1α*), calmodulin (*CAM*), RNA polymerase largest subunit (*RPB1*), and RNA polymerase second largest subunit (*RPB2*) gene regions, were amplified and sequenced. The detail information of corresponding primers were showed as follows: ITS (ITS4: TCCTCCGCTTATTGATATGC; ITS5: GGAAGTAAAAGTCGTAACAAGG) [12], *EF–1α* (EF1: ATGGGTAAGGARGACAAGAC; EF2: GGARGTACCAGTSATCATG) [14], *CAM* (CL1: GARTWCAAGGAGGCCTTCTC; CL2A: TTTTTGCATCATGAGTTGGAC) [15], *RPB1* (Fa: CAYAARGARTCYATGATGGGWC; G2R: GTCATYTGDGTDGCDGGYTCDCC) [16], *RPB2* (5f2: GAYGAYMGWGATCAYTTYGG; 7cr: CCCATRGCTTGYTTRCCCAT) [17]. The PCR programs were set as follows: initial denaturation at 95 °C for 90 s, followed by 35 cycles, at 95 °C for 30 s, annealing for 30 s, and extension at 72 °C for 1 min, as well as terminating with a final extension at 72 °C for 10 min. The annealing temperatures were 55 °C, 56 °C, 55 °C, 58 °C, and 58 °C for ITS, *EF–1α*, *CAM*, *RPB1*, and *RPB2*, respectively. The PCR products were sent to the company (TSINGKE, Beijing, China) for purification and sequencing.

### 2.4. Phylogenetic Analysis

The obtained sequences were analyzed by BLASTn (nucleotide blast) searches. The relevant strains (Table 1) were selected both according to BLAST searches and previous references [18]. The five gene sequences were concatenated and edited manually in MEGA v.7.0.26 [19], and the aligned dataset was deposited in TreeBASE. The maximum likelihood [20] and Bayesian inference (BI) methods were used to phylogenetic analysis the ITS, *EF–1α*, *CAM*, *RPB1*, and *RPB2* combing sequences for *Fusarium incarnatum–equiseti* species complex (FIESC). ML analysis was performed using RAxML (Randomized Axelerated Maximum Likelihood) v.7.2.8 (A. Stamatakis, Heidelberg, Germany) [21]. The branch support was assessed with 1000 replicates. Bayesian inference (BI) analyses were conducted in MrBayes v.3.2.1 (Huelsenbeck J P and Ronquist F, Rochester, USA) by using the Markov chain Monte Carlo (MCMC) algorithm [22]. Mrmodel test v.2.3 (Posada D and Crandall K A, Oxford, England) [23] was used to determine the best fit evolutionary model (GTR + I + G) using the Akaike information criterion (AIC) parameter. Two MCMC chains were run in the random tree, with a total of 1 million generations, sampling once every 100 generations. When the average standard deviation of the separation frequency was less than 0.01, the first 25% of the samples were discarded, and the operation was stopped. The trees were viewed and edited with Figtree v.1.3.1 [19]. The ITS, *EF–1*, *RPB2*, *RPB1*, and *CAM* sequences of strain GZAX 312 were deposited under GenBank numbers ON961780, ON982724, ON982726, ON982728, and ON982722, respectively.

### 2.5. Pathogenicity Test

Pathogenicity was tested on living tobacco leaves and stems. Healthy tobacco were surface-sterilized in 2% sodium hypochlorite and washed 3 times with sterilized distilled water for 2 min before performing the test [12]. The experiment was repeated three times, with at least three plants for each time. For leaves and stems inoculation, the mycelium block (about 6 mm) cultured on the PDA medium was inoculated to the healthy leaves and base of the stalk and wrapped with absorbent cotton and plastic wrap to keep it moisturized. After inoculation, the plants were cultured in a greenhouse (22 °C, under a light/dark period of 12/12 h). Control plants were inoculated with PDA plugs. Lesion was observed daily, and photographic record for 7 days after inoculation. For root inoculation, fresh mycelium blocks were mixed with rye seeds and cultured at 28 °C for 7 days [1]. Then, 15 culture seeds were mixed into the 5 cm surface soil of each pot and cultured at 22 °C after adding sufficient water. Four days later, the healthy tobacco seedlings at 4-leaf stage were planted into the pot and cultured in greenhouse. Seven days later, the disease symptoms of roots were observed. Seedlings planted in sterile soil were used as controls. The pathogen was re-isolated from the inoculation site using PDA medium. The morphological characteristics and RPB2 sequence were compared with original strains.

## 3. Results

### 3.1. Serious Leaf Spot and Blight Disease Was Observed on Field Tobacco Plants

**Figure 1:**
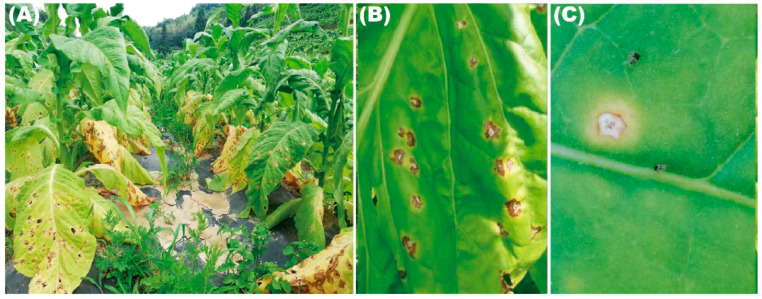
(**A**–**C**) Diseased tobacco leaf symptoms in the field.

The field survey of tobacco disease was conducted in August 2021. During the surveillance, it was normally surrounded by a yellow halo, which appeared on the tobacco leaves, as shown in Figure 1. Surveys indicated a 22 to 35% disease incidence in three counties of Zheng’an and Fenggang in Guizhou province and Shanglin in Guangxi Zhuang Autonomous Region. With the development of the disease, spots were enlarged and concatenated. Severely infected leaves turned out to be blight, then defoliation. In order to determine the causal agents, 35 disease samples were collected from the three counties, and the pathogenic fungi were isolated in the laboratory.

### 3.2. Morphology Characterization Indicated a Fusarium *spp.*

In total, 77 fungal strains were isolated from those 35 samples. Among them, 68 strains showed similar cultural characters, such as the white colony, with cotton and flocculent aerial mycelium. Thus, they seem to be one species and could be the dominant pathogen for tobacco leaf spot and blight disease. Three represent strains (GZAX 307, GZAX 312, and GZAX 402) were randomly selected from all isolated strains for subsequent research. Observation indicated the three strains had same morphological characteristics. Therefore, the strain GZAX 307 was used for the following microscopy visualization and morphology description.

**Figure 2:**
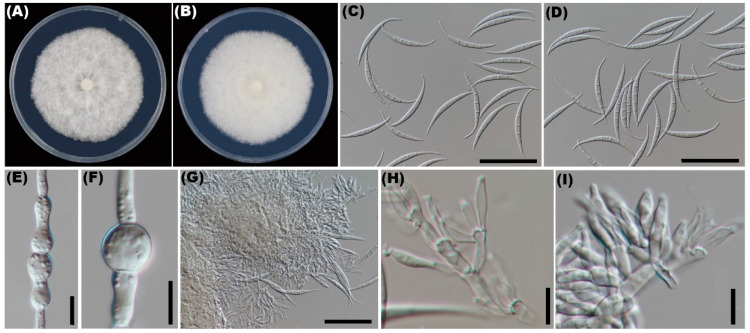
(**A**,**B**) Colony on PDA after incubation for 7 days. (**C**,**D**) Macroconidia. (**E**,**F**) Chlamydospores. (**G**–**I**) Conidiogenous cells form on sporodochia. Bars: (**C**,**D**,**G**) = 50 μm; (**E**,**F**,**H**,**I**) = 10 μm.

The fungal colonies reached 61–62 mm in diam. and were white in color, with cotton flocculent aerial mycelium after 7 days of incubation on PDA (Figure 2A,B). On the surface of the carnation leaves, the sporodochial macroconidia falcate, prominently curved, apical cell papillate to hooked, and basal cell had distinct foot shapes, 4–6 septa, most of which were 5 septa, macroconidia 44–118 × 4–11 μm (Figure 2C,D). Chlamydospores were not observed on PDA; few chlamydospores were produced singly, doubly, or in chains after 2 weeks of CLA under alternating 12 h darkness/12 h fluorescent light at 25 °C (Figure 2E,F). The conidiophores in the sporodochia were variable in length, verticillately branched, and densely packed, with most bearing the apical whorls of three monophialides, sporodochial phialides subulate to subcylindrical, thin-walled, hyaline (Figure 2G–I). No sexual structures were observed. These characteristics suggest the fungus was *Fusarium* sp. [24].

### 3.3. Phylogenetic Analyses Identified a Fusarium ipomoeae Agent

**Figure 3:**
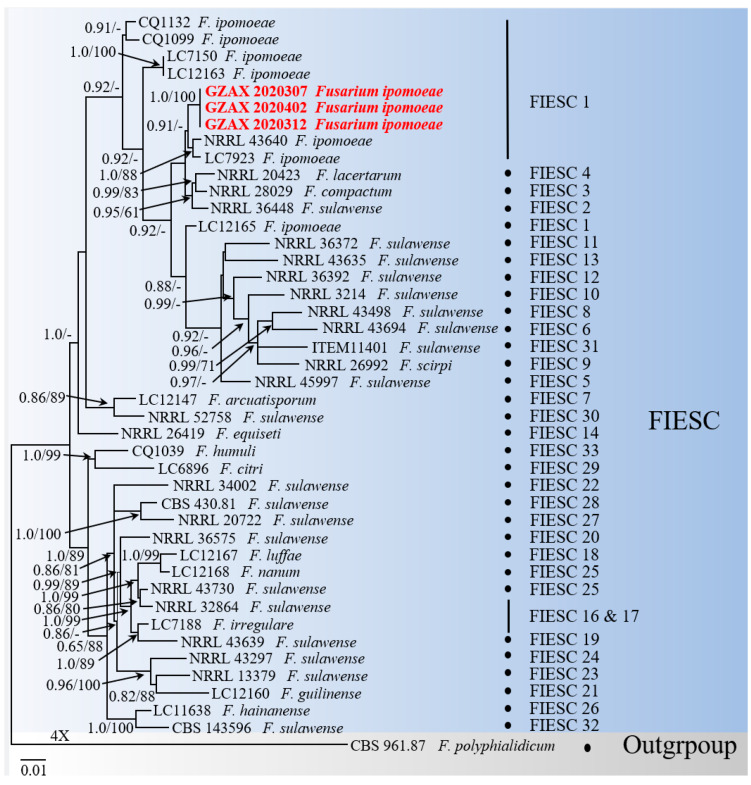
Phylogenetic tree was constructed using strains of *Fusarium incarnatum*–*equiseti* complex (FIESC), based on ITS, *EF–1α*, *CAM*, *RPB1*, and *RPB2* loci. MrBayes posterior probabilities (PP > 65) and ML bootstrap (BS > 60%) support values were showed at the nodes (PP/BS).

To further identify the causal agent, the phylogenetic analysis, based on the combination of five gene locus sequences, were performed (take GZAX 307 as an example). Firstly, the obtained sequences were analyzed on BLASTn, and the results showed that over 99% nucleotide sequence identity with members of the FIESC: the ITS sequence showed 100% identity (488/1132 bp) to the *F. lacertarum* strain NRRL 20423; the *EF–1α* sequence showed 99.56% identity (684/678 bp) to the *F. ipomoeae* strain NRRL 43640; the *RPB2* sequence showed 100% identity (747/839 bp) to the *F. ipomoeae* strain CBS 140909; the *RPB1* sequence showed 99.42% identity (1726/1578 bp) to the *F. sulawense* strain LC12173; and the *CAM* sequence showed 99.28% identity (715/698 bp) to the *F. ipomoeae* strain NRRL 34034. Then, phylogenetic analysis, using concatenated sequences of ITS, *EF1–α*, *RPB2*, *RPB1*, and *CAM*, showed that GZAX 307, GZAX 312, and GZAX 402 clustered monophyletically with strains of *F. ipomoeae* (the relevant strains are shown in Table 1). Therefore, based on the morphological and molecular characteristics, the isolates GZAX 307, GZAX 312, and GZAX 402 were identified as *F. ipomoeae* (Figure 3).

### 3.4. F. ipomoeae Showed Pathogenicity on Leaf and Stem

**Figure 4:**
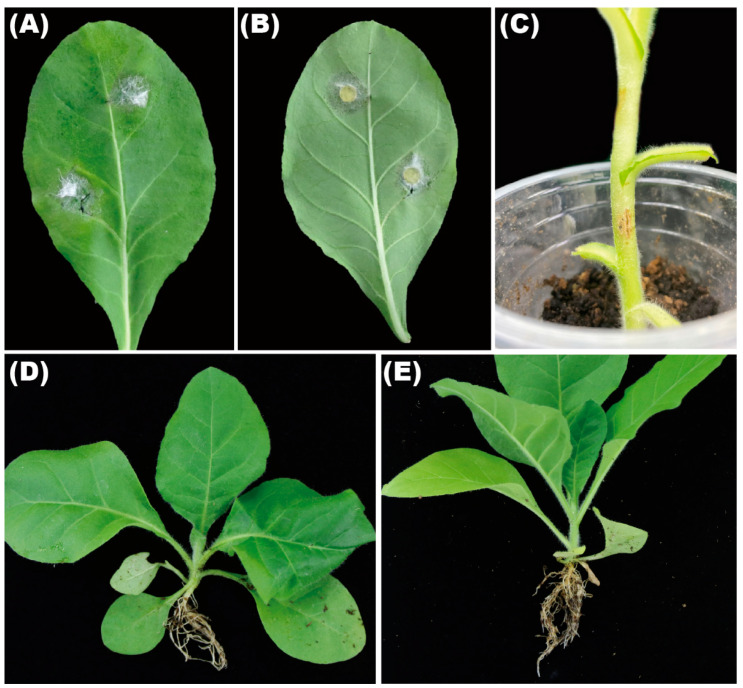
Pathogenicity on tobacco (**A**,**B**) leaves, (**C**) stems, and (**D**) roots. (**E**) The roots of control plant.

To complete the Koch postulates, the strains GZAX 307, GZAX 312, and GZAX 402 were inoculated on the leaves and stems, as well as the roots. The specific morphology and pathogenicity statistics are shown in Table 2. The pathogenicity results were consistent; therefore, the specific description took GZAX 307 as an example. The results of pathogenicity were the same, and strain 307 was taken as an example to show the symptoms. About 7 days post inoculation, white hyphae crawled on the leaves from the inoculation site; meanwhile, small brown spots appeared around the hyphae (Figure 4A,B). The lesion diameter can reach 42.6 ± 3.02 mm. The pathogen was re-isolated from the inoculated sites and further validated as the same fungus through morphological and phylogenetic analyses. Light brown spots appeared at the inoculation site of tobacco stem after 7 days (Figure 4C). After inoculation, no obvious pathogenicity symptoms occurred in the roots of the tobacco, compared with the control (Figure 4D,E).

## 4. Discussion

Dried and fermented tobacco leaves are raw resources for tobacco industry and product manufacturing [2]. Thus, owning to its important commercial values, this annual, leafy, solanaceous plant was planted globally; therefore, it creates jobs, increases incomes, maintains tax revenues, and sustains trade surpluses [2]. However, the field production of this cash crop is frequently seriously threatened by many fungal pathogens [7,25]. Thus, the safe and sustainable production of tobacco leaves required control of fungal diseases. Additionally, better understanding of the infection of pathogenic fungi is better for the control of the disease.

Previously, in order to know more about the incidence of fungal pathogens in the process of tobacco cultivation, we went to the tobacco-producing areas of Guizhou province and Guangxi Zhuang autonomous region to investigate. During the process of our investigation, we found that the symptoms were irregular brown spots, comprising whitish center, normally surrounded by yellow halo appearing on tobacco leaves. The disease was not deadly, but it seriously affected the quality and greatly reduced the economic value of tobacco. Further investigation revealed that a prevalence of the tobacco leaf spot was 22 to 35% in the field. In order to investigate the pathogen, we collected the samples of this disease from three random sites; through isolation and identification, we found that the dominant pathogen was *Fusarium ipomoeae* (Figure 2 and Figure 3).

Several studies have found that *F*. *ipomoeae* is associated with plant growth process, which serves as a causal pathogen that significantly affects the quality and quantity of products. Previously, *F*. *ipomoeae* have been reported to cause leaf spots on peanuts [26] and *Bletilla striata* [27] in China. However, to our knowledge, this is the first report worldwide of *F*. *ipomoeae* causing leaf spots on tobacco. Compared with the strain found in *Bletilla striata*, the colony and spore morphology in this study was consistent, but there were some differences in the spore sizes. In peanuts, the macroconidia were 4–7 septate, and 3.3–4.5 × 18.5–38.1 μm in size. In *bletilla striata*, the macroconidia were 3–5 septate, and 3.3–4.5 × 18.5–38.1 μm in size. In this study, macroconidia were 4–6 septa, most of which were 5 septa, with the macroconidia 44–118 × 4–11 μm in size. Czapek-Dox agar was used for determining the species strains from peanuts and PDA medium for strains from *Bletilla striata*, which were different from the CLA medium used in this study. However, CLA medium is commonly used to observe the spore morphology of *Fusarium* spp. [28]. The difference in spore size may also be related to host or cultural conditions, and it is necessary to study further.

Pathogenicity assays showed that leaf spot symptoms appeared on the inoculated leaves after 4 days post inoculation in *Bletilla striata* [27], and symptoms similar to those in the field were observed on leaves after 10 days inoculation in peanuts [26]. In the present study, leaf spot symptoms appeared on the inoculated tobacco leaves after one day. The pathogenicity of this pathogen to tobacco leaves was moderate and consistent with the field diseased symptom. A single inoculation site is not large, but when it spreads to the entirety of the tobacco leaves, tobacco production takes a pathogenic blow. Therefore, we should pay close attention to this pathogen in further disease control.

## Figures and Tables

**Table 1:** Strains used in the phylogenetic analyses and their GenBank accession numbers.

Species	Phylogenetic Species	Strain	Host	Location	ITS	*EF–1*	*CAM*	*RPB2*	*RPB1*
*F. ipomoeae*	FIESC 1	CQ1099	*Rhododendron pulchrum* leaf	Jiangsu, China	MK280853	MK289573	MK289715	MK289727	MK289861
		LC7923	*Capsicum* sp.	Shandong, China	MK280800	MK289635	MK289688	MK289789	MK289853
		CQ1132	*Vinca major* leaf	Jiangsu, China	MK280854	MK289574	MK289716	MK289728	MK289862
		NRRL 43640 = UTHSC 04–123	Dog nose	Texas, America	GQ505756	GQ505667	GQ505578	GQ505845	HM347191
		LC12165 = CGMCC3.19496 (T)	*Ipomoea aquatica* leaf	Fujian, China	MK280832	MK289599	MK289704	MK289752	MK289859
		LC7150	Bamboo	Jiangxi, China	MK280818	MK289627	MK289678	MK289781	MK289852
		LC12163	*Hibiscus syriacus*	Fujian, China	MK280790	MK289597	MK289700	MK289750	MK289857
		**GZAX 402**	**Tobacco**	**Guizhou, China**	**OP454871**	**OP432881**	**OP432880**	**OP432883**	**OP432882**
		**GZAX 307**	**Tobacco**	**Guizhou, China**	**ON961779**	**ON982723**	**ON982721**	**ON962725**	**ON962727**
		**GZAX 312**	**Tobacco**	**Guangxi, China**	**ON961780**	**ON982724**	**ON982722**	**ON982726**	**ON982728**
*F. sulawense*	FIESC 2	NRRL 36448 = CBS 384.92	*Phaseolus vulgaris* seed	Sudan	GQ505741	GQ505652	GQ505564	GQ505830	–
*F. compactum*	FIESC 3	NRRL 28029 = CDC B–3335	Human eye	California, America	GQ505691	GQ505602	GQ505514	GQ505780	HM347150
*F. lacertarum*	FIESC 4	NRRL 20423 = IMI 300797 (T)	Lizard skin	India	GQ505682	GQ505593	GQ505505	GQ505771	JX171467
*F. sulawense*	FIESC 5	NRRL 45997 = UTHSC 04–1902	Human sinus	Colorado, America	GQ505761	GQ505672	GQ505583	GQ505850	–
		NRRL 34035 = UTHSC 91–569	Human sinus	Colorado, America	GQ505726	GO505637	GQ505549	GQ505815	–
*F. sulawense*	FIESC 6	NRRL 43694 = CDC 2006743607	Human eye	Texas, America	GQ505757	GQ505668	GQ505579	GQ505846	HM347193
*F. arcuatisporum*	FIESC 7	LC12147 = CGMCC3.19493 (T)	*Brassica campestris* pollen	Hubei, China	MK280802	MK289584	MK289697	MK289739	MK289799
*F. sulawense*	FIESC 8	NRRL 43498	Human eye	Pennsylvania, America	GQ505747	GQ505658	——	GQ505836	HM347181
*F. sulawense*	FIESC 30	NRRL 52758 = ARSEF 4714	*Prosapia* nr. *bicincta* on Cynodon	Costa Rica	JF740925	JF740833	——	JF741159	——
*F. scirpi*	FIESC 9	NRRL 26992 = CBS 610.95	Soil	France	GQ505681	GQ505592	GQ505504	GQ505770	——
*F. sulawense*	FIESC 31	ITEM11401	*Avena sativa*	Canada	——	LN901578	LN901594	LN901611	——
*F. sulawense*	FIESC 10	NRRL 3214 = FRC R–6054, 7.13 MRC	Unknown	Unknown	GQ505676	GQ5O5587	GQ505499	GQ505765	——
*F. sulawense*	FIESC 13	NRRL 43635 = UTHSC 06–638	Horse	Nebraska	GQ505751	GQ505662	GQ505573	GQ505840	HM347188
*F. sulawense*	FIESC 12	NRRL 36392 = CBS 259.54	Unknown plant seedling	Germany	GQ505739	GQ505650	GQ505562	GQ505828	——
*F. sulawense*	FIESC 11	NRRL 36372 = CBS 235.79	Air	Antilles, Netherlands	GQ505738	GQ505649	GQ505561	GQ505827	——
*F. equiseti*	FIESC 14	NRRL 26419 = CBS 307.94, BBA 68556 (NT)	Soil	Germany	GQ505688	GQ505599	GQ505511	GQ505777	——
*F. irregulare*	FIESC 15	LC7188 = CGMCC3.19489 (T)	Bamboo	Guangdong, China	MK280829	MK289629	MK289680	MK289783	MK289863
*F. sulawense*	FIESC 16 & 17	NRRL 32864 = FRC R–7245	Human	Texas, America	GQ505702	GQ505613	GQ505525	GQ505791	HM347160
		NRRL 43730 = CDC 2006743605	Contact lens	Mississippi, America	EF453193	GQ505669	GQ505580	GQ505847	——
*F. luffae*	FIESC 18	LC12167 = CGMCC3.19497 (T)	Luffa aegyptiaca	Fujian, China	MK280852	MK289569	MK289711	MK289723	MK289870
*F. sulawense*	FIESC 19	NRRL 43639 = UTHSC 04–135	Manatee	Florida, America	GQ505755	GQ505666	GQ505577	GQ505844	HM347190
*F. sulawense*	FIESC 20	NRRL 36575 = CBS 976.97	*Juniperus chinensis* leaf	Hawaii, America	GQ505745	GQ505656	GQ505568	GQ505834	——
*F. sulawense*	FIESC 22	NRRL 34002 = UTHSC 95–1545	Human ethmoid sinus	Texas, America	GQ505715	GQ505626	GQ505538	GQ505804	HM347165
*F. sulawense*	FIESC 23	NRRL 13379 = FRC R–5198, BBA 62200	*Oryza sativa*	India	GQ505680	GQ505591	GQ505503	GQ505769	——
*F. sulawense*	FIESC 24	NRRL 43297 = W. Elmer 22	*Spartina rhizomes*	Connecticut, America	GQ505746	GQ505657	GQ505569	GQ505835	——
*F. sulawense*	FIESC 27	NRRL 20722 = IMI 190455	*Chrysanthemum* sp.	Kenya	GQ505684	GQ505595	GQ505507	GQ505773	——
*F. guilinense*	FIESC 21	LC12160 = CGMCC3.19495 (T)	*Musa nana* leaf	Guangxi, China	MK280837	MK289594	MK289652	MK289747	MK289831
*F. sulawense*	FIESC 28	CBS 430.81 = NRRL 28577	Grave stone	Romania	GQ505692	GQ505603	GQ505515	GQ505781	——
*F. sulawense*	FIESC 32	CBS 143596	*Stereum irsutum*	Iran	LT970815	LT970779	LT970732	LT970751	——
*F. nanum*	FIESC 25	LC12168 = CGMCC3.19498 (T)	*Musa nana* leaf	Guangxi, China	GQ505697	GQ505608	GQ505520	GQ505786	——
*F. hainanense*	FIESC 26	LC11638 = CGMCC3.19478 (T)	*Oryza* sp. stem	Hainan, China	MK280836	MK289581	MK289657	MK289735	MK289833
*F. citri*	FIESC 29	LC6896 = CGMCC3.19467 (T)	*Citrus reticulata* leaf	Hunan, China	MK280803	MK289617	MK289668	MK289771	MK289828
*F. humuli*	FIESC 33	CQ1039 = CGMCC3.19374 (T)	*Humulus scandens* leaf	Jiangsu, China	MK280845	MK289570	MK289712	MK289724	MK289840
*F. polyphialidicum*	——	CBS 961.87	Plant debris	South Africa	GQ505763	GQ505674	GQ505585	GQ505852	——

**Table 2:** Morphology characterization and pathogenicity of *F*. *ipomoeae*.

Species (Strain)	Location	Colonies (mm)	Conidia		Pathogenicity on Tobacco Leaf (mm)		On Stem (mm)		On Root (mm)
			Body (μm)	Septa	Wounded	Unwounded	Wounded	Unwounded	
*F*. *ipomoeae* (GZAX 307)	Zheng’an in Guizhou province	61.5 ± 0.5	44–118 × 4–11	4–6	37 ± 2	34 ± 3	18 ± 2	12 ± 6	—
*F*. *ipomoeae* (GZAX 312)	Shanglin in Guangxi Zhuang autonomous region	62 ± 1	47–123 × 2–9	4–6	35 ± 1	32 ± 2	20 ± 4	11 ± 5	—
*F*. *ipomoeae* (GZAX 402)	Fenggang in Guizhou province	62.5 ± 1	42–120 × 4–14	4–6	33 ± 4	34 ± 3	17 ± 2	10 ± 5	—

## Data Availability

The ITS, *EF–1*, *RPB2*, *RPB1*, and *CAM* sequences of strain GZAX 307 were deposited under GenBank numbers ON961779, ON982723, ON982725, ON982727, and ON982721, respectively. The ITS, *EF–1*, *RPB2*, *RPB1*, and *CAM* sequences of strain GZAX 312 were deposited under GenBank numbers ON961780, ON982724, OM982726, ON982728, and ON982722, respectively.
